# Editorial: Vital role of innate immunity in cancer immunotherapy

**DOI:** 10.3389/fimmu.2025.1594009

**Published:** 2025-05-21

**Authors:** Sibusiso Luthuli, Jiazhu Wu, Xin Zhou

**Affiliations:** ^1^ Department of Hematology, First Affiliated Hospital of Nanjing Medical University, Nanjing, China; ^2^ Department of Oncology, First Affiliated Hospital of Nanjing Medical University, Nanjing, China; ^3^ Department of Oncology, The Affiliated Suqian First People’s Hospital of Nanjing Medical University, Suqian, China

**Keywords:** innate immunity, complement, biomarkers, cancer, neutrophil extracellular traps, tumor microenvironment, immunotherapy, toll-like receptor

**Figure 1:**
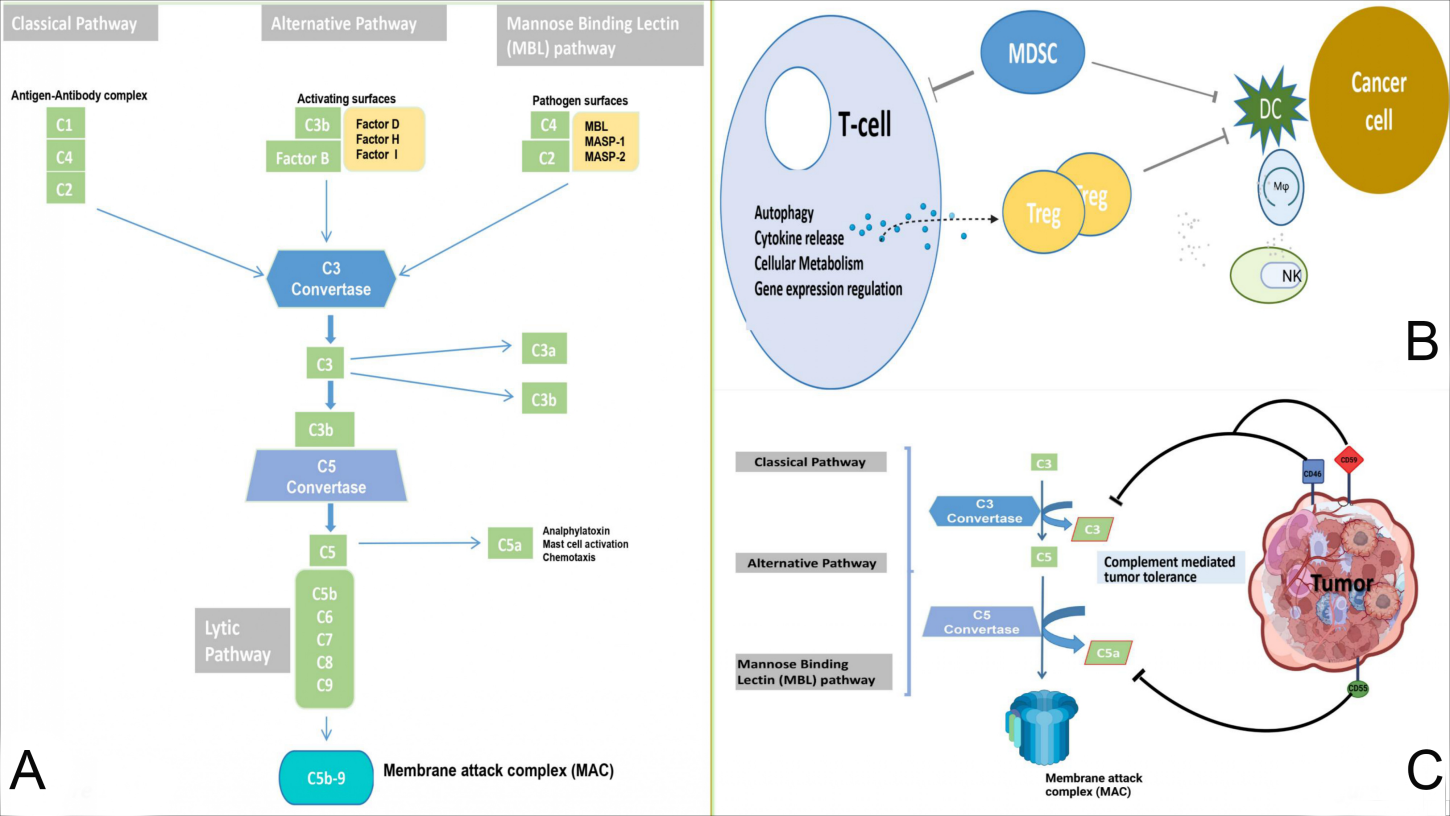
**(A)**: Normal anti-tumor immunity and complement pathways. The classical, lectin, and alternative pathways are used to form C3 convertase and C5 convertase, which results in the cleavage of C5 into C5a and C5b. This process facilitates the formation of MAC and cytotoxicity against cancer cells. **(B, C)**: The complement system mediates tumor immune tolerance. Complement components and inhibitory receptors are produced by cancer cells to facilitate their growth and suppress the immune response. These components contribute to the establishment of an immunosuppressive environment, promote Treg differentiation, and influence T cells. Additionally, they impede the function of antigen-presenting cells, recruit MDSCs, and influence macrophage polarization and NK cell function. Mφ, macrophage; MDSCs, myeloid-derived suppressor cells; DC, dendritic cells; NK, natural killer cell.

The complement system, a cornerstone of innate immunity, acts as a ‘double-edged sword’ in cancer immunotherapy (1). As highlighted by Yang et al., it can both combat malignancy by enhancing the efficacy of therapeutic antibodies and promote tumor progression by shaping the tumor microenvironment (TME). This duality depends on factors such as cell type-specific complement distribution, signaling balance within the TME, and tumor proliferation dynamics (**Figure 1**). For example, in lung cancer, cytotoxic T cells efficiently eliminate tumor cells expressing strong antigens. In contrast, in pancreatic cancer, the same antigens may accelerate disease progression due to a lack of dendritic cells needed for T-cell activation (2). Therefore, the complement system may contribute, directly or indirectly, to the development of drug resistance and reduce the effectiveness of cancer therapies.

This context-dependent plasticity of innate immune cells underscores the need to unravel their dual mechanisms. Such insights could drive innovations in biomarkers and therapies, i.e., unlocking novel T cell-based immunotherapies that interconnect innate and adaptive immunity. These cells produce pro-inflammatory cytokines (e.g., IFN-γ, IL-12, IL-18) and exhibit innate-like cytotoxicity while retaining adaptive features via T-cell receptors (TCRs) (3). The development of immunotherapy has transformed the clinical management of cancer, positioning it as a key strategy that activates innate immunity and promotes durable, protective T-cell responses. Immunotherapies, including cell-based treatments, immune checkpoint inhibitors (ICIs), and tumor vaccines, have demonstrated substantial anticancer activity (4). Despite these advances, long-term cancer control remains challenging. Further research is needed to overcome current limitations and to identify new therapeutic targets that can expand the effectiveness and applicability of immunotherapy.

Decroos et al. investigated CD8+ T cells in leukemia patients who achieved treatment-free remission (TFR) despite the persistence of residual cancer cells. Their study revealed a strong correlation between elevated natural killer (NK) cell ativity—key anti-tumor effectors—and TFR. They also identified a subset of innate CD8+ T cells co-expressing Eomesodermin and inhibitory markers (KIR/NKG2A) that exhibited enhanced cytotoxicity through perforin upregulation. In addition, reduced PD-1 expression on effector T cells was found to serve as a predictive biomarker for TFR in chronic myeloid leukemia (CML). Although these findings are promising, their applicability to other hematological malignancies beyond CML remains limited, particularly when targeting ICIs such as anti-PD-1. A more established role of innate CD8+ T cells has been observed in solid tumors, including ovarian cancer (5). Integrating past and current insights from leading journals such as Frontiers in Immunology may stimulate new ideas and collaborative initiatives to advance translational research and further elucidate the critical role of innate immunity in cancer immunotherapy.

In clinical practice, cancer patients often develop resistance to therapy, highlighting the need for improved immunotherapeutic strategies. Although immune checkpoint therapies activate immune cells by modulating cytokines and chemokines, they can also promote tumorigenesis through changes in the TME and can cause systemic toxicity. An ideal cancer immunotherapy should stimulate both systemic innate and adaptive immune responses while minimizing systemic damage. Therefore, targeting both innate and adaptive mechanisms is critical to establishing a durable and effective anti-tumor immune response (6).

The immune system eliminates disease by recognizing common pathogen components and activating innate and adaptive immune pathways, which are also essential for cancer immunosurveillance, often mediated by cytokines and chemokines. Xiao et al. conducted a comprehensive review of publications from 2006 to 2024, identifying emerging strategies involving neutrophil extracellular traps (NETs) and bacterial therapies. NETs, once thought to be solely protective against infection, are now implicated in cancer progression. This review highlights the role of NETs in promoting an immunosuppressive TME, positioning NET-targeted therapies as a promising frontier in cancer immunotherapy. Historically, systemic bacterial administration has faced major toxicity challenges. For example, Coley demonstrated that intravenous (i.v.) administration was effective but often caused uncontrollable toxicity. Encouragingly, modern approaches have shown greater promise, as illustrated by the FDA-approved Bacillus Calmette-Guérin (BCG) vaccine, derived from attenuated *Mycobacterium bovis*, which has demonstrated clinical efficacy in the treatment of bladder cancer (7–9).

Newman proposed the development of a systemically administered, attenuated, and killed bacteria-based multi-immune receptor agonist for anti-tumor immunotherapy. The study identified Toll-like receptors (TLRs) and their related agonists, with particular emphasis on the potent TLR4 agonist, lipopolysaccharide (LPS), which constitutes approximately 75% of the outer membrane of Gram-negative bacteria (G-NB). LPS was shown to serve as both a critical active component and a major contributor to the dose-limiting intravenous toxicity associated with G-NB. The study further demonstrated that attenuated, stabilized, intact bacterial products derived from non-pathogenic G-NB retained approximately 96% of their endotoxin activity. Notably, Decoy10, a formulation with agonist activity for TLR2, TLR4, TLR8, TLR9, NOD2, and STING, showed reduced intravenous toxicity in both mice and rabbits while preserving the ability to stimulate cytokine and chemokine secretion by human immune cells *in vitro*, comparable to unprocessed parental bacterial cells.

Using bacteria as delivery systems for cancer therapy offers a promising approach to achieve long-term protection through the induction of immunological memory, thereby supporting sustained cancer control. Nevertheless, further research and development are required to fully realize the potential of this strategy in immunotherapy.

These studies underscore the critical role of innate immunity in shaping the tumor microenvironment and advancing immunotherapy. They have identified additional targets that may overcome therapeutic resistance and improve treatment efficacy. The discovery of novel targets involving CD8+ T cells, NETs, and attenuated bacterial therapies may drive future research in this field. Ongoing investigations into the complexity of the innate immune system in cancer biology suggest that new therapeutic strategies may further enhance immunotherapy approaches. Moving forward, it will be essential to conduct research that builds on these findings to translate our expanding understanding of innate immunity and cancer immunotherapy into effective clinical applications.
